# Obtaining the Most Accurate, Explainable Model for Predicting Chronic Obstructive Pulmonary Disease: Triangulation of Multiple Linear Regression and Machine Learning Methods

**DOI:** 10.2196/58455

**Published:** 2024-08-29

**Authors:** Arnold Kamis, Nidhi Gadia, Zilin Luo, Shu Xin Ng, Mansi Thumbar

**Affiliations:** 1 Brandeis International Business School Brandeis University Waltham, MA United States

**Keywords:** chronic obstructive pulmonary disease, COPD, cigarette smoking, ethnic and racial differences, machine learning, multiple linear regression, household income, practical model

## Abstract

**Background:**

Lung disease is a severe problem in the United States. Despite the decreasing rates of cigarette smoking, chronic obstructive pulmonary disease (COPD) continues to be a health burden in the United States. In this paper, we focus on COPD in the United States from 2016 to 2019.

**Objective:**

We gathered a diverse set of non–personally identifiable information from public data sources to better understand and predict COPD rates at the core-based statistical area (CBSA) level in the United States. Our objective was to compare linear models with machine learning models to obtain the most accurate and interpretable model of COPD.

**Methods:**

We integrated non–personally identifiable information from multiple Centers for Disease Control and Prevention sources and used them to analyze COPD with different types of methods. We included cigarette smoking, a well-known contributing factor, and race/ethnicity because health disparities among different races and ethnicities in the United States are also well known. The models also included the air quality index, education, employment, and economic variables. We fitted models with both multiple linear regression and machine learning methods.

**Results:**

The most accurate multiple linear regression model has variance explained of 81.1%, mean absolute error of 0.591, and symmetric mean absolute percentage error of 9.666. The most accurate machine learning model has variance explained of 85.7%, mean absolute error of 0.456, and symmetric mean absolute percentage error of 6.956. Overall, cigarette smoking and household income are the strongest predictor variables. Moderately strong predictors include education level and unemployment level, as well as American Indian or Alaska Native, Black, and Hispanic population percentages, all measured at the CBSA level.

**Conclusions:**

This research highlights the importance of using diverse data sources as well as multiple methods to understand and predict COPD. The most accurate model was a gradient boosted tree, which captured nonlinearities in a model whose accuracy is superior to the best multiple linear regression. Our interpretable models suggest ways that individual predictor variables can be used in tailored interventions aimed at decreasing COPD rates in specific demographic and ethnographic communities. Gaps in understanding the health impacts of poor air quality, particularly in relation to climate change, suggest a need for further research to design interventions and improve public health.

## Introduction

### Background

Lung disease is a severe problem in the United States. According to the Centers for Disease Control and Prevention (CDC), asthma is responsible for at least 3000 deaths per year and chronic obstructive pulmonary disease (COPD) is responsible for at least 150,000 deaths per year. COPD is a progressive lung disease, encompassing chronic bronchitis and emphysema, which is characterized by airflow limitation and breathing difficulties. Asthma and COPD can co-occur (asthma-COPD overlap), with increased risk of mortality [1] and diminished disease-related quality of life [2]. This is from a variety of factors, some under individual control, such as cigarette smoking, and others not under individual control, such as ambient air pollution.

Cigarette smoking has been trending downward in recent years, thanks in part to public health advertisement campaigns. Nevertheless, air quality can be dangerously poor at times, which exacerbates lung health problems [3], and the impacts can be particularly acute in populations considered vulnerable. Technologically, there are tools that help individuals avoid poor air quality. For example, there are mobile phone apps that track air quality. They notify their owners on days when air quality is dangerously poor, advising them to stay indoors or avoid strenuous outdoor exercise. The effectiveness of such apps is ambiguous thus far [4,5].

The rest of the paper is organized as follows. We first review prior work regarding the possible factors contributing to COPD in adults. We then describe our methods, including data sources for the variables of interest and descriptive statistics. Following this, we will describe and interpret the results of our multiple linear regression (MLR) and machine learning (ML) models. We conclude by describing the overall research contributions as well as limitations and future directions.

### Prior Work

There is substantial literature on factors contributing to COPD, including a wide variety of environmental, economic, and demographic variables; the etiology of COPD is multifactorial, with smoking being the most well-known contributing factor. Furthermore, the combination of environmental pollutants and cigarette smoke has shown synergistic effects, accelerating the decline in lung function and worsening COPD [6,7]. In addition, occupational exposures, for example, to coal dust, arsenic, or diesel fumes, or to home exposures, such as gas stoves, wood stoves, kerosene heaters, and fireplaces, contribute to overall COPD outcomes. When combined with persistent ambient air pollution, the risk and severity of COPD will likely increase [8].

Pollutants and copollutants are associated with decreased lung function and can lead to COPD. The loss can range from mild, such as allergies, to severe, that is, mortality. Air quality varies widely throughout the United States because of pollutants and copollutants, and climate change may be worsening it, particularly for populations considered vulnerable [9]. Health disparities due to poor quality air and other stressors are well known [10-12]. Ambient air pollution in poorer neighborhoods tend to be exacerbated by additional copollutants, heat stress, and aeroallergens. Air quality index (AQI) includes the totality of pollutants and copollutants.

ML methods have been applied increasingly to public health and medical problems. For example, ML has been used to support the public health response to COVID-19 through surveillance, case identification, contact tracing, and evaluating interventions [13]. ML methods have been used as a supportive tool to recognize cardiac arrest in emergency calls [14]. In that study, Zicari et al [14] developed a general protocol with a collaborative team to ensure that the ML tool was domain- and context-sensitive as well as abiding by ethical guidelines, thus obtaining trustworthiness. ML has been also used to improve early and accurate stroke recognition during emergency medical calls [15].

ML methods have been used to study COPD, in particular. For example, ML methods have been used to develop a prediction system using lifestyle data, environmental factors, and patient symptoms for the early detection of acute exacerbations of COPD within a 7-day window [16]. Another study on acute exacerbations of COPD compared several ML methods and found that a decision tree classifier was best for assessing patient severity and guiding treatment strategy [17]. In another study, to improve mortality prediction from COPD, a random forest was used to identify the most important imaging features [18]. Gradient boosted trees (GBTs) have been used to predict lung function values from computed tomography images obtained from patients with COPD and those without COPD [19]. Deep learning has been effective in analyzing images diagnostic of COPD [20]. Finally, research using a generalized linear model found a complex relationship between rural living and COPD-related outcomes in US veterans [21]. Thus, a variety of ML models have been successfully applied for use in public health scenarios in general and COPD in particular. The one that ultimately works best in a given situation depends on many factors.

Different races and ethnicities may have different baseline rates of disease due to various factors, including historical misdiagnosis and mistreatment of various racial or ethnic groups, which leads to differential outcomes [22]. There may be outcome, equity, and counseling differences by gender as well as race or ethnicity in the diagnosis and treatment of COPD [23,24].

We had three general expectations of COPD in our models:

1. Cigarette smoking will have the highest impact on COPD rates.
2. AQI will have a strong impact on COPD rates.
3. There will be differences in COPD rates based on racial or ethnic demographics.

## Methods

### Overview

This paper used MLR and ML methods to predict COPD at the core-based statistical area (CBSA) level [25]. At the time of this study, there were 388 metropolitan and 541 micropolitan statistical areas in the United States. The data sources were obtained from data repositories of 3 official US agencies, specifically from the CDC. We gathered, integrated, and checked them for data quality. By combining different variables from this variety of data sources, we aimed to obtain a uniquely high accuracy model, while simultaneously reducing biases or flaws that may be attributable to individual data sources. We further checked for missing values (ie, NULL or NA) in every variable. We checked for data correctness by checking the plots of the distributions for every variable, looking for impossible or outlying values. Table 1 shows the data sources used.

**Table 1:** Data sources.

Source	Reference
National Center for Health Statistics	[26]
Chronic Disease Indicators data	[27]
US Chronic Disease Indicator, stratification values	[28]

Data were collected for all CBSAs that were available from 2016 to 2019. All data obtained from the CDC were contributed voluntarily at the individual level and aggregated to remove all personally identifiable information [29].

The COPD rates are for 2019, whereas all the predictor variables are averaged over the timespan from 2016 to 2018. As such, the models obtained are predictive over time. The data collection result was 517 (56%) of the 929 CBSAs, with proportionally more from the 388 metropolitan statistical areas than from the 541 micropolitan statistical areas. The response variable is the percentage of the CBSA having COPD. We modeled all factors as random variables directly contributing to COPD, which is measured as the proportion (percentage) of the population having COPD. Race or ethnicity was also modeled as percentage of the population rather than as categorical variables. All variables in Table 2 are averaged as mean, except for household income, which was averaged as median.

**Figure 1:**
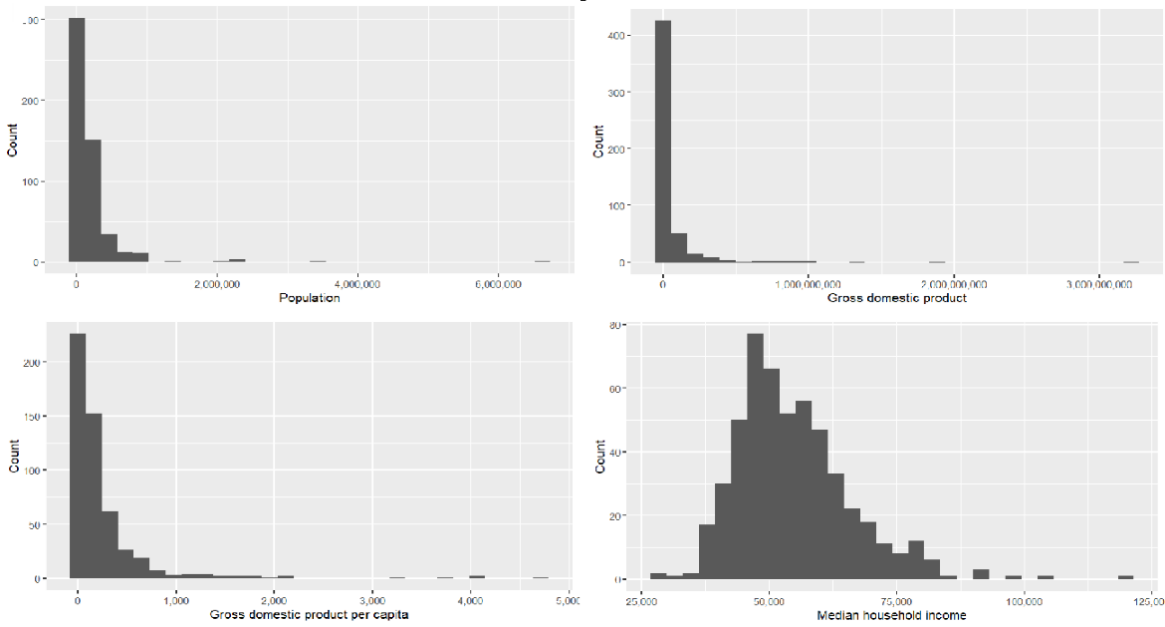
Population, gross domestic product (GDP), GDP per capita, and median household income.

In Figure 1, we observe that some variables (ie, population, gross domestic product [GDP], GDP per capita, and median household income) are skewed in their distribution.

**Table 2:** Main variables and descriptive statistics and average within core-based statistical areas.

	Years	Values, median (IQR)	Values, mean (SD)	Values, range
Population (n)	2016-2018	96,811 (48,763-180,484)	191,892 (408,308)	7351-6,633,096
GDP^a^ (US $)	2016-2018	13,126,907 (2,562,704-39,046,120)	64,223,036 (212,975,821)	447,355-3,218,209,695
Median household income (US $)	2016-2018	52,632 (46,867-60,494)	54,736 (11,319)	27,842-119,332
GDP per capita (US $)	2016-2018	100.07 (47.83-277.17)	253.77 (479)	16.86-4731.50
Air quality index	2016-2018	38.67 (34.00-43.00)	38.02 (10)	9.00-95.00
Smoking rate	2016-2018	17.12 (15.33-19.28)	17.29 (3)	8.41-29.59
Poverty rate (all ages)	2016-2018	13.80 (10.92-17.12)	14.36 (4)	3.87-35.56
Unemployment rate	2016-2018	4.52 (3.67-5.43)	4.71 (2)	1.97-20.93
Education rate	2016-2018	22.91 (17.94-27.96)	24.22 (8)	8.77-65.75
White (%)	2016-2018	87.6 (78.6-92.8)	84.6 (0.129)	22.1-100
Black (%)	2016-2018	4 (1.5-12.5)	9.3 (0.124)	0.3-100
AI or AN^c^ (%)	2016-2018	0.7 (0.4-1.7)	2 (0.044)	0.1-45.9
Asian (%)	2016-2018	1.6 (0.9-3)	2.8 (0.041)	0.2-42.8
NH or PI^d^ (%)	2016-2018	0.1 (0.1-0.2)	0.3 (0.01)	0.0-12.9
Hispanic (%)	2016-2018	7 (3.9-14.9)	13.3 (0.164)	0.9-95.5
COPD^b^ rate (%)	2019	6.7 (5.7-7.9)	6.871 (1.511)	3.2-15

^a^GDP: gross domestic product.

^b^COPD: chronic obstructive pulmonary disease.

^c^AI or AN: American Indian or Alaska Native.

^d^NH or PI: Native Hawaiian or Pacific Islander.

**Figure 2:**
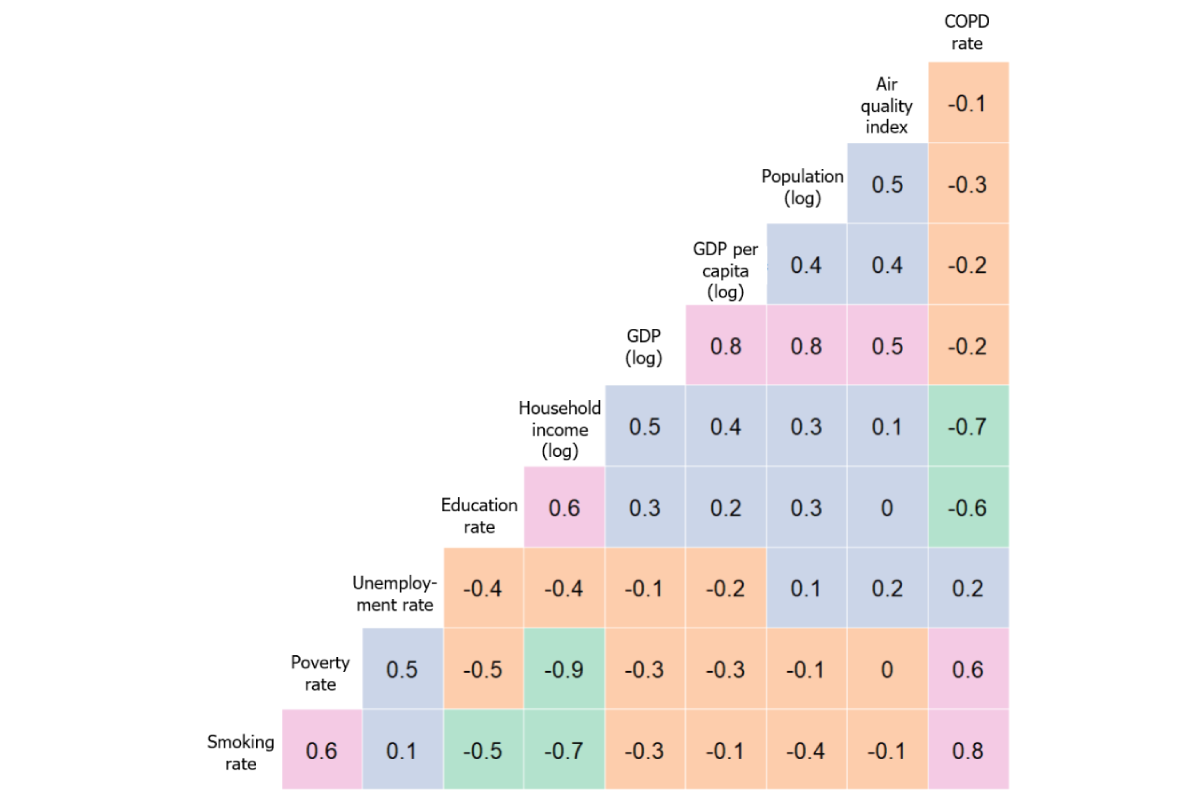
Correlations among main variables. COPD: chronic obstructive pulmonary disease; GDP: gross domestic product.

Therefore, we made a log transformation of these variables (ie, logPopl, logGDP, logGDPpc, and logHHI) to make them less skewed, and we show a heat map of correlations of them with the other variables in Figure 2.

We see a range of correlations, from very negative (green) to negative (orange) to positive (purple) to very positive (pink). In the rightmost column, we see the correlations between the response variable, COPD rate, and the other variables, ranging from very positive (smoking rate) to moderately positive (poverty and unemployment rates) to moderately negative (education and logged household income) to slightly negative (log of GDP, log of population, log of GDP per capita, and AQI). Given these correlations, we are likely to find good predictive models, but we need to check for multicollinearity in any linear model that we identify.

To understand and model COPD, one has to consider the consistently largest contributing factor: cigarette smoking. Research tends to either control for cigarette smoking or exclude it entirely. In this paper, we chose to include cigarette smoking, accounting for it in our models, but also to examine other factors to compare the magnitudes of influence among the various factors. We aimed to model a variety of factors, including cigarette smoking, to arrive at the model that predicts COPD with the greatest accuracy.

### Statistical Analysis

#### Overview

Our MLR baseline model in R (version 4.2.3) yielded the output in Table 3, which is sorted by absolute value of the *t* value, from high to low.

**Table 3:** Multiple linear regression.

	Estimate	SE	*t* test (*df*=503)	*P* value
(Intercept)	32.4000	2.930	11.065	<.001
Smoking_Rate	0.2570	0.015	16.635	<.001
Log_HH_Income	−2.8100	0.264	−10.638	<.001
Hispanic_percentage	−2.3900	0.234	−10.249	<.001
Education_Rate	−0.0334	0.005	−6.627	<.001
AI_or_AN_percentage^a^	−2.9000	0.778	−3.726	<.001
Black_percentage	−1.2700	0.356	−3.558	<.001
NH_or_PI_percentage^b^	−15.8000	4.430	−3.558	<.001
Log_GDP	0.0741	0.035	2.126	.034
White_percentage	−0.7380	0.358	−2.060	.04
Unemployment_Rate	0.0456	0.023	1.993	.047
Asian_percentage	2.4800	1.250	1.988	.047
Log_Population	0.0899	0.055	1.626	.105
Air_Quality_Index	0.0003	0.003	0.098	.922

^a^AI_or_AN: American Indian or Alaska Native.

^b^NH_or_PI: Native Hawaiian or Pacific Islander.

The model has residual SE 0.658 on 503 *df*. The multiple *R*^2^ is 0.8152 and adjusted *R*^2^ is 0.8105. The *F*-statistic is 170.7 on 13 and 503 *df* (*P*<.001). The variance inflation factors were checked, with all values <5 indicating low multicollinearity.

There are 7 predictors of high statistical significance: smoking rate, Black percentage, Native Hawaiian or Pacific Islander percentage, American Indian or Alaska Native percentage, education rate, Hispanic percentage, and log of household income. Smoking rate has a positive association with COPD, with every additional percentage increase associated with a 0.257% increase in the COPD rate. The other 6 highly significant predictors have a negative association. Every percentage increase in the log of household income lowers the COPD rate by 2.81%. The Hispanic percentage is nearly as strong; every percentage increase corresponds to a drop of 2.39% in COPD rate. American Indian or Alaska Native is a bit stronger in its coefficient estimate; every percentage point increase corresponds to a drop of 2.9% in COPD rate. Every percentage point increase in Native Hawaiian or Pacific Islanders corresponds to a drop of 15.8%, which is much stronger. Every percentage point increase in Black percentage corresponds to a drop of 1.27% in COPD rate. Education rate has a strongly statistically significant relationship, but a small percentage point impact: every percentage increase corresponds to a decrease of 0.0334% in COPD rate. The remaining 4 predictors—White percentage, GDP (logged), unemployment rate, and Asian percentage—are far less statistically significant and, therefore, should be interpreted with caution.

Linear models are simpler than ML models, and they are sometimes perfectly adequate for explaining a phenomenon. They are easier to interpret, communicate, and implement as new policy. They make statistical assumptions, which can be verified. Linear regression is certainly a good place to start. However, we argue that one should not stop there because an ML model can capture substantial variance from nonlinear relationships (if there are any) in the data and thus produce a more accurate model. By capturing additional variance, the model can capture subtler effects and relationships due to interactions, context, and tipping points. This is crucial because public health practice tends to use simple if-then rules, that is, decision trees. ML models can add nuance to those decision trees based on the captured nonlinearities. Although an adjusted *R*^2^ of 0.8105 looks quite strong, we can perhaps do better with ML methods [18-21].

The 7 ML methods evaluated in this paper are lasso regression, ridge regression, generalized additive model, support vector machine, artificial neural network, random forest, and GBT. These methods were selected for their known strengths in minimizing errors of bias or errors of variance, that is, their ability to fit data well on test data without overfitting. They also represent the range of algorithms commonly used in ML prediction, from methods established in classical statistics to more modern methods derived from computer science. They are commonly used because they are accurate and well understood. Trying a variety of methods is a common practice because the different methods make different statistical assumptions, which may enhance or inhibit optimal performance. All methods were available as R packages for R (version 4.2.3). We summarize each method in terms of its main pros and cons:

#### Lasso Regression (L1 Regularization)

Lasso regression is an MLR method that incorporates regularization to perform variable selection. It minimizes the sum of squared errors between predicted and actual values, while adding a penalty term based on the absolute value of coefficients multiplied by a tuning parameter. Doing so shrinks some coefficients to exactly 0, effectively performing feature selection by excluding less important variables from the model. This reduces model complexity and minimizes multicollinearity. This is a standard refinement of MLR (R package glmnet).

#### Ridge Regression (L2 Regularization)

Ridge regression is an MLR technique that adds a penalty term to the objective function to reduce the coefficients of less important predictors and guard against overweighting the most important predictors. While it retains all predictors in the model, ridge regression can help improve the robustness of the model in the presence of correlated predictors by reducing multicollinearity. This is a standard refinement of MLR (R package ridge).

#### Generalized Additive Model

The generalized additive model is a nonparametric generalization of MLR, which allows for nonlinear terms and coefficient regularization while maintaining interpretability. Each term is a function of X_n_ rather than simply a numeric coefficient multiplied with X_n_. As with MLR, all the terms are added together. Although overfitting can occur, regularization and cross-validation help to minimize it (R package mgcv).

#### Support Vector Machine

Support vector machine is a technique that transforms the data into a high-dimensional variable space using a kernel function, fitting a function that best fits the data while allowing a certain margin of error (epsilon) and maintaining robustness against outliers. Epsilon tubes can provide a visual representation of the model’s uncertainty. Points within the tube are considered well predicted, while those outside represent errors. A regularization parameter controls the trade-off between accuracy and complexity (R package e1071).

#### Artificial Neural Network

Artificial neural network is a generalization of MLR with hidden layers of nodes between input and output nodes; it may result in overfitting. Depending on the number of hidden layers, nodes per layer, and the activation function used to convert inputs to outputs, an arbitrarily complex model can be fit. This can be thought of as a simplified version of a human brain, in which input and output nodes are separated by ≥1 layers of hidden nodes. Prediction error causes the weights of the hidden nodes to be adjusted until minimal error is achieved (R package neuralnet).

#### Random Forest

Random forest is an ensemble technique to fit a large number of a bootstrap-sampled aggregation (bagging) of trees by considering a random subset of variables at each tree split. Intuitively, a random forest is a blending of a large number of decision trees, the “wisdom of the forest.” The random subset of variables restriction is done to prevent strong variables from dominating the weaker variables. A random forest tends to perform very well but is difficult to interpret (R package RandomForest).

#### Gradient Boosted Trees

GBT is an ensemble of sequential trees that focuses on the errors of the previous tree. It is able to find interaction effects implicitly. It uses gradient descent search to rapidly minimize error via an arbitrary, differentiable loss function. It uses many trees to help ensure that the local minimum error found is the global minimum. Intuitively, this builds a strong predictive model by combining many weak models, each correcting the errors of the previous one (R package XGBoost).

Our ML approach followed best practices. We randomly partitioned the data set into train (311/517, 60%), cross-validate (103/517, 20%), and test (103/517, 20%) subsets. We checked for outliers, multicollinearity, and target leakage to ensure valid models [30].

### Ethical Considerations

This research did not involve human subjects at the individual level and therefore did not require institutional review board approval. Our data were collected from CDC sources at the level of CBSA. All sources were free of personal identifying information, because the CDC is legally required to ensure the protection of the data. All data were collected and aggregated in a non–personal identifying information manner. The results of our analysis do suggest communicating with different racial and ethnic groups differently, tailoring the implications directly to patients as well as indirectly to their families, communities, and health care providers in a race- or ethnicity-sensitive manner.

## Results

In Table 4, we describe the results of the ML models of COPD by various accuracy metrics. For the accuracy metrics, we used 3 standard measures of predictive accuracy in addition to variance explained (adjusted *R*^2^): root mean square error (RMSE), mean absolute error (MAE), and symmetric mean absolute percentage error (SMAPE) [31,32]. We performed a grid search over all the main numeric parameters for a given method to find the optimal combination of parameter values [33]. A grid search tries all combinations of parameters from a minimum to a maximum value by some step size. Those minimum, maximum, and step sizes are determined from typical default values and best practices. The best metrics in Table 4 are indicated by italics.

**Table 4:** Machine learning models versus multiple linear regression.

Method	Adjusted *R*^2^	Root mean square error				Mean absolute error				Symmetric mean absolute percentage error		
		Train	CV^a^	Test	Train		CV	Test	Train		CV	Test
Gradient boosted tree (XGBoost, loss function=least squares, learning rate=0.05, and maximum tree depth=10)	0.857	0.550	0.598	0.557	0.433		0.445	*0.456* ^b^	6.473		6.543	*6.956*
Support vector machine (Nystroem kernel and loss function=Poisson deviance)	*0.858*	0.555	*0.558*	*0.556*	0.435		*0.434*	0.462	6.515		*6.443*	6.989
Random forest (maximum trees=500, maximum depth=none, and maximum leaves=100)	0.836	*0.534*	0.614	0.596	*0.420*		0.462	0.479	*6.315*		6.819	7.339
Neural network (2 layers: 512, 512 units; regularization via random dropout rate=0.05 and activation function=prelu)	0.845	0.601	0.609	0.580	0.455		0.467	0.468	6.856		6.928	7.182
Generalized additive model (learning rate=0.3, maximum bins=100, and loss function=least squares)	0.822	0.629	0.658	0.621	0.515		0.508	0.488	7.619		7.502	7.212
Ridge regression	0.810	0.589	0.618	0.641	0.467		0.483	0.527	6.986		7.346	7.986
Lasso regression	0.758	0.750	0.778	0.724	0.585		0.593	0.597	8.425		8.544	8.824
Multiple linear regression	0.811	0.620	0.699	0.749	0.474		0.548	0.591	7.205		8.403	9.666

^a^CV: cross-validation.

^b^Values in italics represent the best metrics.

The ML methods were superior to MLR on most metrics. Support vector machine was the best on adjusted *R*^2^ and RMSE, slightly superior to GBT, but GBT was superior by a larger margin on MAE and SMAPE. Therefore, we chose GBT as the best overall method. In Multimedia Appendix 1, we show the variable importance plot for the GBT model. Variable importance plots are a common first way to peer inside a “black-box method” and understand the relative importance of the variables used within it [34].

The top five variables in terms of impact were (1) smoking rate and (2) household income, followed by (3) American Indian or Alaska Native percentage, (4) education rate, and (5) unemployment rate. Black percentage was sixth, Hispanic percentage was seventh, and there was only a small impact from the remaining variables: White percentage, AQI, Asian percentage, Native Hawaiian or Pacific Islander percentage, population, and GDP. Relative to the MLR, smoking rate, household income, education rate, and Black percentage remained the same in terms of rank importance. Hispanic percentage dropped from third to seventh rank; American Indian or Alaska Native percentage rose from fifth to third rank; and unemployment rate rose sharply, from 10th to 5th in importance. Native Hawaiian or Pacific Islander percentage dropped sharply, from 7th to 11th in rank.

**Figure 3:**
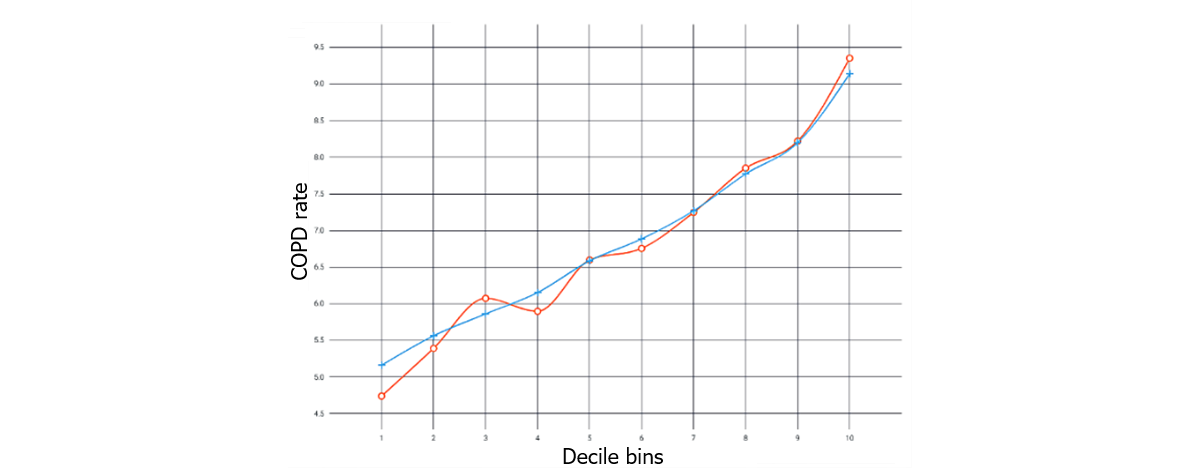
Lift plot showing chronic obstructive pulmonary disease rate as a function of 10 decile bins; predicted values are in blue and actual values are in red. COPD: chronic obstructive pulmonary disease.

**Figure 4:**
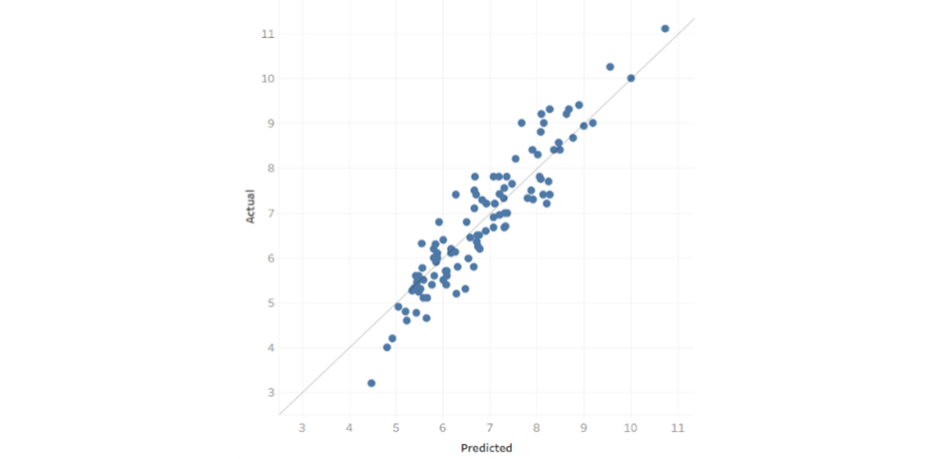
Prediction residuals.

Figure 3 shows the lift plot, and Figure 4 shows the predictive residual plot. The lift plot shows observations sorted by predicted value deciles. The ratio of the observed outcome to the expected outcome was calculated and plotted. The predictive residual plot shows the differences between observed and predicted values.

**Figure 5:**
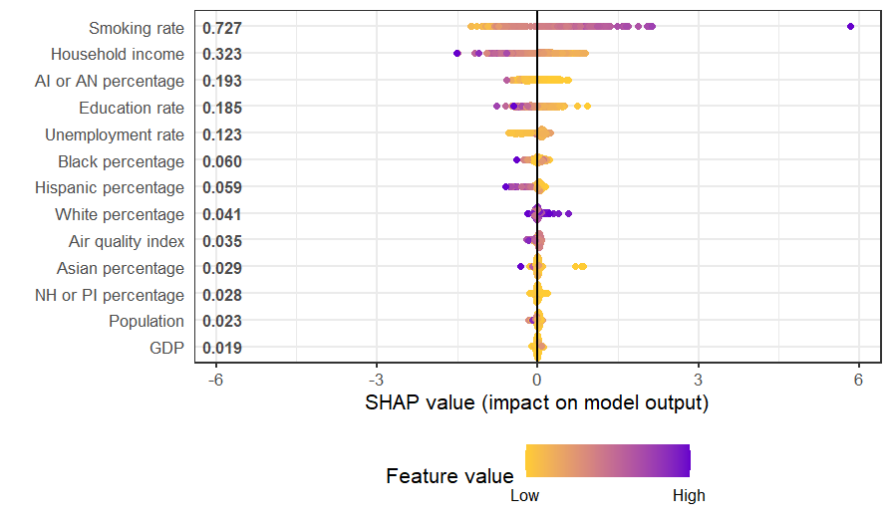
SHAP (Shapley additive explanations) values for all features (variables). AI: American Indian; AN: Alaska Native; GDP: gross domestic product; NH: Native Hawaiian; PI: Pacific Islander.

In addition to the variable importance plot, other plots were used to gain an understanding of ML models: local interpretable model-agnostic explanations (LIME) models and SHAP (Shapley additive explanations) plots [35-37]. We chose SHAP plots because they are based on a cooperative game-theoretical foundation, showing every combination of the variables in the model and how they work together to predict the outcome variable. Figure 5 shows the SHAP plot for all the GBT’s variables.

**Figure 6:**
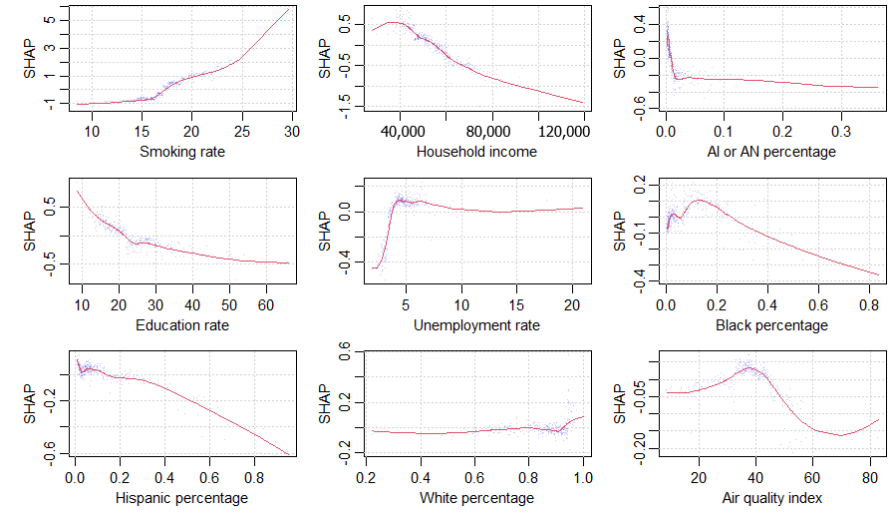
SHAP (Shapley additive explanations) plots for the 9 most important variables. AI: American Indian; AN: Alaska Native.

The top 5 variables (smoking rate, household income, American Indian or Alaska Native percentage, education rate, and unemployment rate) have substantially more impact on COPD percentage than the remaining variables. We show the top 5 variables as well as the next 4 as individual SHAP plots of the GBT in Figure 6. All 9 plots show significant nonlinearities.

Smoking had the greatest impact: as the smoking rate increased, the COPD rate also rose substantially, following a steeply curved, nearly exponential relationship. Median household income had the second highest impact, an almost linear (and negative) relationship. The greater the household income, the lower the COPD rate. This could indicate better insurance coverage, better health care access, higher quality health care (ie, prevention or treatment), lower occupational exposure, or lower home exposure (eg, gas stoves). The next variable was American Indian or Alaska Native percentage, indicating a negative but nonlinear relationship with COPD rate: a steep drop followed by a gradual tapering. This represents a significant protective influence shown for the American Indian or Alaska Native community, which has not yet been noted in the literature.

The next variable, education rate had a negative, curvilinear relationship. The more educated the population, the lower the COPD rate. The explanation could be similar to that of income: better insurance coverage or health care access, better quality of health care, lower occupational exposure, or lower home exposure [38]. The next variable was unemployment rate, with a sharply positive but flat relationship with COPD rate. The next variable was Black percentage, with an initial positive relationship with COPD rate but then a reversal to a negative, linear relationship.

The next variable, Hispanic percentage, showed a negative linear relationship with COPD rate. This represents a significant protective influence shown for people in the Hispanic community, which is consistent with the literature [39-45]. The next variable was White percentage, showing a slightly negative relationship with COPD rate. Finally, the last variable was AQI (higher value being worse), which shows an initial positive relationship with COPD rate, peaking around 38. This may be a critical point, after which people take precautions not to be exposed to the low-quality air.

## Discussion

### Principal Findings

We had three general expectations, which were largely met:

1. The impact of cigarette smoking was the largest in all models.
2. The AQI had an impact in the best ML model, but it was smaller than expected.
3. There were substantial racial or ethnic differences, particularly among American Indian or Alaska Native, Black, and Hispanic communities.

Consistent with the literature, we found that smoking remains the most significant risk factor for COPD, with research consistently demonstrating a strong association between smoking status and COPD prevalence. In our MLR, we found that smoking rate is the strongest predictor of COPD rate. We found the same result in our GBT but also found that the smoking rate has a curvilinear, almost exponential, relationship with COPD. The Rotterdam study, a large-scale population-based cohort study, found that current and former smokers had a substantially higher risk of developing COPD compared to never smokers [46]. A nationwide population-based cohort study in South Korea demonstrated that smoking cessation after COPD diagnosis was associated with lower all-cause and cause-specific mortality [47].

Notably, 3 of the 4 next most important variables, in terms of impact in our GBT, are socioeconomic variables: household income (rank 2), education rate (rank 4), and unemployment rate (rank 5). In the MLR, we found that household income (logged) had the second highest impact. In the GBT, household income had the second highest impact, but the tipping point was around US $40,000, after which higher income had a linear, negative relationship with COPD. Education rate had a strongly negative, curvilinear relationship with COPD. Unemployment rate had a sharply positive relationship with COPD, but then peaked at 5% unemployment, after which it plateaued.

These results are largely consistent with the literature on socioeconomic factors and smoking behavior, suggesting an indirect relationship with COPD via smoking. A study examining smoking among adolescents in 6 European cities found that disposable income was positively associated with smoking [48]. Conversely, lower socioeconomic status was associated with higher COPD prevalence because in addition to lower education and income, there may be environmental pollutants, occupational hazards, or barriers to COPD screening, diagnosis, and treatment [49]. In contrast with the literature, our SHAP plots show mostly nonlinear relationships with COPD. Household income showed a tipping point at US $40,000, after which the negative relationship with COPD was nearly linear.

Ethnic or racial variables accounted for 3 of the top 7 variables in the GBT: American Indian or Alaska Native percentage (rank 3), Black percentage (rank 6), and Hispanic percentage (rank 7). The greater the size of those minority populations, the lower the COPD rate. Our SHAP plots show significant tipping points (nonlinearities) for American Indian or Alaska Native percentage and Black percentage and a mostly linear relationship for Hispanic percentage. Consistent with the literature, all 3 variables show a strongly negative association with COPD.

The regression and GBT models show that in addition to strongly protective impacts for lower cigarette smoking and higher household income, there are protective impacts for larger American Indian or Alaska Native and Hispanic populations as well as a nonlinear impact on larger Black populations. Higher education rate and lower unemployment rate are also protective, whereas AQI shows mixed effects. These results have implications for private health care practitioners, public health care officials, and health care policy makers who aim to reduce COPD rates. Such policies and programs should not assume high digital literacy [50,51]. System designers could use SMS text messaging, social media, and interactive voice response systems. This would be appropriate for those with lower household income or lower education levels. To design culturally appropriate visual cues and messaging to different racial or ethnic groups, members of the various communities should be included in the design process [52,53]. In sum, the user interface should exhibit high ease-of-use—using gamification, storytelling, and peer support—consistent with cultural norms.

Several studies have identified ethnic and racial disparities in COPD prevalence and risk among smokers. One study found that racial and ethnic minority individuals, particularly African Americans and Hispanics, had a lower prevalence of airflow obstruction than non-Hispanic White individuals, even after adjusting for smoking status and other risk factors [54]. This finding was supported by another study that observed lower COPD risk in ethnic minority groups compared to White individuals, despite similar smoking intensities [55]. A larger minority population means a larger peer support network for prevention and cessation of smoking and a larger peer community to recommend COPD screening, diagnosis, and treatment, which is particularly useful in a health care system that has implicit racial or ethnic bias [50,56].

There are varying levels of patient trust and implicit bias in health care practitioners themselves [57], which contributes to health outcome differences. From a population communication perspective, messaging regarding the risks of COPD—particularly the avoidance or cessation of cigarette smoking—should be sensitive to community context, engaging trusted local authorities to optimize the chances of patient engagement [58]. Health care practitioners could partner with trusted local authorities and community leaders regarding smoking prevention and cessation as well as respiratory health in general to decrease COPD risk. Health care practitioners and educators should communicate to different populations in culturally sensitive ways [59,60].

Educational materials and behavior change strategies may need to be customized according to different risk factors, beliefs, preferences, and technographics of different subpopulations [50,51]. On a basic level, people with lower levels of education or household income could be directed via phone geolocation to their local health care and to their community leaders for in-person guidance and support. Local leaders could then inform them about local smoking cessation programs and apps or websites that monitor air quality in their community. Trusted local authorities are helpful entry points in those communities, after which peer support and network effects spread the information.

AQI was not significant in the MLR, but it was significant in the GBT, albeit not as strongly as we expected. It could be that the AQI is more of a diffuse, macrolevel environmental factor that fluctuates over time, making some CBSAs worse on average, but with wide volatility, for example, as weather and wind directions change [61,62]. Therefore, AQI could have more of an indirect or interaction effect with other variables. Combining campaigns on smoking prevention with campaigns on air quality could create a holistic public health strategy, particularly—as our findings suggest—in communities considered vulnerable, that is, communities with lower education, higher unemployment, and lower household income. Subsidies for households in communities considered vulnerable to convert to more efficient, cleaner home heating and cooling methods would improve their home’s air quality at a lower cost [63]. Research suggests that engaging communities in targeting their air quality issues can lead to more positive outcomes in both air quality and public health [64-66].

There is a small but growing body of research that uses ML models in health care and medicine. There is recognition that the models can be highly accurate, but there is no consensus yet on how to interpret the results in a way that meshes seamlessly with clinical practice. The following examples provide an overview.

Elshawi et al [67] compared model-agnostic explanations using 2 techniques, LIME and Shapley values, to interpret a ML model for predicting hypertension risk. LIME uses small subsets of the data, which may be idiosyncratic, to provide intuitive explanations, that is, rules. Shapley values are more theoretically sound and global, using all the available data, and are, therefore, less idiosyncratic than LIME, but they do not provide LIME’s simple, linear explanations [67].

Hakkoum et al [68] conducted an extensive literature review of ML interpretability in medicine published between 1994 and 2020. The review found that there was no consensus on evaluation metrics or frameworks to assess the quality and utility of the interpretability methods [68]. The highest performing ML models did not translate easily into clinical rules.

Meng et al [69] reviewed the interpretability and fairness evaluation of deep learning models on MIMIC-IV data set, a large, publicly available benchmark for developing and evaluating the interpretability of high-performing ML models that use sensitive demographic features. The review found that existing interpretation methods, for example, variable importance rankings, provide partial explanations without fully elucidating the model’s complex decision logic.

In sum, there is no consensus on the best way to interpret high-performing ML models in health care. There are always trade-offs between accuracy and interpretability or explainability. We chose to use Shapley values because they represent the frontier in explainability, and they are similar to interpreting a multiple regression, interpreting 1 variable at a time, without the assumptions of linear models. In addition, Shapley values allow for nonlinear relationships between each independent (predictor) variable and the dependent variable. Variable importance plots in conjunction with Shapley values help us to identify the most important variables and characterize their relationships with COPD.

Our best MLR model had variance explained of 81.1%, MAE of 0.591, and SMAPE of 9.666. Our best ML model was the GBT, with variance explained of 85.7%, MAE of 0.456, and SMAPE of 6.956. The GBT explains most of the variance—4.6% more than the best MLR—with far less predictive error. The GBT’s SMAPE (6.956) was 28% lower than that of the MLR’s SMAPE (9.666). Similarly, the GBT’s RMSE was 26% lower than the MLR’s RMSE, and its MAE was 23% lower than that of the MLR. Real-world predictive accuracy should be similar to that found in the test data set because the test data were never used in the GBT’s model development.

Our GBT performed strongly on the test data, with very little performance deterioration on the test data versus performance on the training and validation data. This demonstrates that the GBT model does not overfit the data. To interpret the GBT, we used a variable importance plot [34,70,71] and SHAP plots [72,73]. SHAP plots are useful for interpreting the strength of the pairwise relationships between predictor variable and COPD rate, showing the added nuances of the curvilinear plots. By doing so, we rendered transparent the “black-box model” [74-76], thus preserving interpretability and actionability, in addition to adding nonlinear nuance.

### Limitations and Future Directions

This research has a few limitations. The data were obtained from 517 (56%) of the 929 CBSAs. We assumed that this was an adequate sample and that the remaining CBSAs that did not report the data were similar to those that did. Alternatively, it could be that the CBSA that did not report COPD rates did so because the rates were low, that is, COPD was not considered a major problem by the local public health officials. Data covering additional demographic variables, such as gender and age, in addition to occupational exposures and physical exercise, could be gathered [77-79]. Future research could develop separate models stratified by demographic variables such as race or ethnicity, assuming there are sufficient data for each categorical class. There could also be geopolitical variations in terms of population density as well as demographics, psychographics [80], and technographics [81,82].

Future data collection could focus on understanding racial or ethnic disparities. By collecting data more intensively from the minority populations, we could go deeper into understanding how their rates of COPD drop so dramatically. Is it related to active peer recommendations for better self-care in a predominantly White health care system and population? Is it related to successfully tailored smoking prevention or cessation programs? Data pertaining to answering these more specific questions could be collected to enhance our understanding of how best to tailor communications to different demographic or ethnographic groups.

All our models were structured as direct effects. We applied MLR and ML methods with data from CBSAs, which have significant variation in terms of health care access and quality. Using these models as a foundation, we should recognize the interconnectedness (ie, direct, indirect, and interactive) of pollutants and copollutants to fully understand COPD’s complex etiology. Future research could model interaction, moderating, or mediating effects, perhaps with a structural equation model, to identify the direct and indirect effects of COPD, for example, showing how asthma may lead to COPD or to asthma-COPD overlap [77].

There are many research knowledge gaps in the health impacts of extreme air pollution, including the effects of interactions between temperature and air pollution on respiratory health due to climate change [83]. Future research directions could focus on modeling the direct and indirect links between environmental exposures and COPD. On the basis of those results, we could design interventions, such as air quality warning systems, to mitigate their impact. The findings would underscore the opportunities for public health regulations, public-private sector partnerships, private company entrepreneurship, and global initiatives to reduce environmental exposures.

Greenhouse gas emissions may exacerbate overall air quality [84-88], contributing indirectly to COPD. Future research could collect data on new, additional variables pertaining to climate change [89]. Wildfires, which are increasingly common, produce more carcinogens in the air, including high levels of particulate matter. This can directly decrease air quality or copollute with other ambient pollutants [90]. These problems have been shown to increase the odds of lung cancer [91], and it is plausible that they can also contribute to COPD.

The association between COPD and environmental pollutants, including tropospheric ozone, nitrogen dioxide, sulfur dioxide, and occupational exposures, has been extensively investigated [8,91-94]. Coarse, fine, and ultrafine particulate matter have been studied extensively and linked to systemic oxidative stress, inflammation [95], atherosclerosis [96], and mortality [97] in the United States [98,99] and China [100-102]. Tropospheric ozone exposure by itself has been linked to impaired lung function and increased COPD-related hospital admissions [103-105]. Similarly, elevated levels of nitrogen dioxide and sulfur dioxide, which are common in cities and industrial work sites, have been linked to an increased risk of COPD in the general population [106,107] and older adults [108]. In sum, data pertaining to ambient pollution, for example, particulate matter, sulfur dioxide, and carbon monoxide, could be useful additional copollutant data to include in future models [6,86-88,91,109-111].

### Conclusions

Our novel contributions in this paper include the following: (1) integration of multiple publicly available CDC data sources, (2) development of highly accurate models using linear and nonlinear methods, and (3) interpretation of the variable impacts for the best model. Smoking was the number 1 variable impacting the COPD rate, which was expected. Household income was the second most influential predictor variable. Four economic factors spanned the full range of influence, from large (household income) to moderate (education rate) to small (unemployment rate and GDP). The race or ethnicity variable also had a range of impacts, from moderately high (American Indian or Alaska Native percentage) to moderate (Black or Hispanic percentage) to small (White, Asian, or Native Hawaiian or Pacific Islander percentage).

This research demonstrates the power of ML methods in general and a GBT, which produced a highly accurate model of COPD rates. The computational complexity of a GBT enables it to obtain high accuracy, but health care policy makers may be reluctant to adopt it unless they can obtain a rule-based explanation. Furthermore, clinicians typically want to be able to explain, justify, and communicate results to others in an intuitive manner. Finally, there may be legal, auditing, or regulatory requirements concerning transparency. If the method is audited, and it cannot be clearly explained, there may be serious legal or financial consequences [72]. Consequently, it is important to have explainable models to open the “black box,” rendering them interpretable and actionable [75,76]. This research shows that it is possible to do so.

## Appendix

Multimedia Appendix 1 Importance of model variables for gradient boosted tree.

## Abbreviations

AQI: air quality index
CBSA: core-based statistical area
CDC: Centers for Disease Control and Prevention
COPD: chronic obstructive pulmonary disease
GBT: gradient boosted tree
GDP: gross domestic product
LIME: local interpretable model-agnostic explanations
MAE: mean absolute error
ML: machine learning
MLR: multiple linear regression
RMSE: root mean square error
SHAP: Shapley additive explanations
SMAPE: symmetric mean absolute percentage error
