# Supplementary figures and images for: Obtaining the Most Accurate, Explainable Model for Predicting Chronic Obstructive Pulmonary Disease: Triangulation of Multiple Linear Regression and Machine Learning Methods
Source: JMIR AI. 2024 Aug 29;3:e58455. doi: 10.2196/58455 (PMC11393512; doi:10.2196/58455)

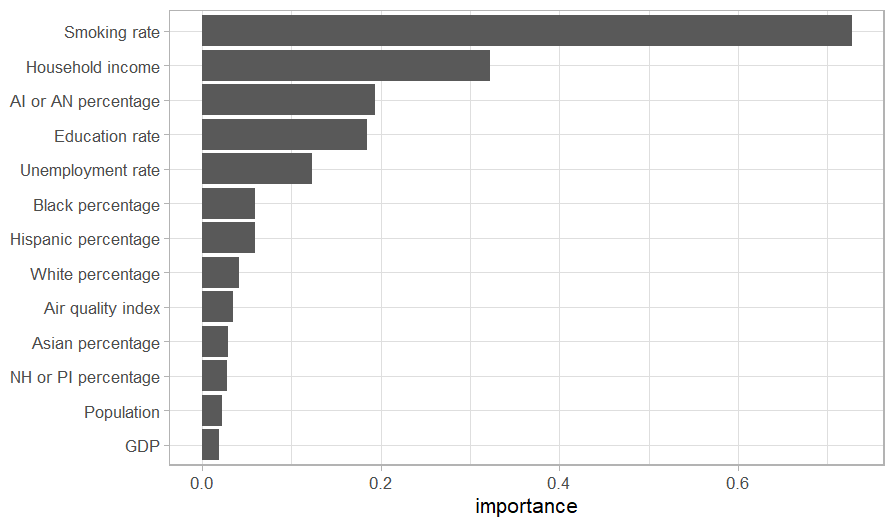

Supplement: Multimedia Appendix 1 [file ai_v3i1e58455_app1.png]
